# The impact of accessibility and service quality on the frequency of patient visits to the primary diabetes care provider: results from a cross-sectional survey performed in six European countries

**DOI:** 10.1186/s12913-020-05421-0

**Published:** 2020-08-26

**Authors:** Uwe Konerding, Tom Bowen, Sylvia G. Elkhuizen, Raquel Faubel, Paul Forte, Eleftheria Karampli, Tomi Malmström, Elpida Pavi, Paulus Torkki

**Affiliations:** 1 Trimberg Research Academy, University of Bamberg, 96045 Bamberg, Germany; 2 Department of Psychology and Psychotherapy, Witten/Herdecke University, Alfred-Herrhausen-Straße 50, 58448 Witten, Germany; 3 The Balance of Care Group, Camden Cottage, Bennett’s Lane, Bath, BA1 5JX UK; 4 Institute of Health Policy & Management, Erasmus University Rotterdam, PO Box 1738, 3000 Rotterdam, DR The Netherlands; 5 Department of Physiotherapy, University of Valencia, Valencia, Spain; 6 Joint Research Unit in Biomedical Engineering (IIS La Fe- Universitat Politècnica de València), Valencia, Spain; 7 Department of Health Economics, National School of Public Health, 196 Alexandras Ave, 115 21 Athens, Greece; 8 Department of Industrial Engineering and Management, Aalto University, Espoo, Finland, PO Box 15500, 00076 Aalto, Finland; 9 Present address: Department of Public Health, Faculty of Medicine, University of Helsinki, P.O. BOX 00020, 00014 Helsingin yliopisto, Finland

**Keywords:** Travel distance, Travel time, In-practice waiting time, Provider-patient communication, Health care provider, Visit, Accessibility to care, Type 2 diabetes

## Abstract

**Background:**

Visits to the primary diabetes care provider play a central role in diabetes care. Therefore, patients should attend their primary diabetes care providers whenever a visit is necessary. Parameters that might affect whether this condition is fulfilled include accessibility (in terms of travel distance and travel time to the practice), as well as aspects of service quality (for example in-practice waiting time and quality of the provider’s communication with the patient). The relationships of these variables with the frequency of visits to the primary diabetes care provider are investigated.

**Methods:**

The investigation is performed with questionnaire data of 1086 type 2 diabetes patients from study regions in England (213), Finland (135), Germany (218), Greece (153), the Netherlands (296) and Spain (71). Data were collected between October 2011 and March 2012. Data were analysed using log-linear Poisson regression models with self-reported numbers of visits in a year to the primary diabetes care provider as the criterion variable. Predictor variables of the core model were: country; gender; age; education; stage of diabetes; heart problems; previous stroke; problems with lower extremities; problems with sight; kidney problems; travel distance and travel time; in-practice waiting time; and quality of communication. To test region-specific characteristics, the interaction between the latter four predictor variables and study region was also investigated.

**Results:**

When study regions are merged, travel distance and in-practice waiting time have a negative effect, travel time no effect and quality of communication a positive effect on visit frequency (with the latter effect being by far largest). When region specific effects are considered, there are strong interaction effects shown for travel distance, in-practice waiting time and quality of communication. For travel distance, as well as for in-practice waiting time, there are region-specific effects in opposite directions. For quality of communication, there are only differences in the strength with which visit frequency increases with this variable.

**Conclusions:**

The impact of quality of communication on visit frequency is the largest and is stable across all study regions. Hence, increasing quality of communication seems to be the best approach for increasing visit frequency.

## Background

Global diabetes prevalence in 2019 is estimated to be 9.3% (463 million people), rising to 10.2% (578 million) by 2030 and 10.9% (700 million) by 2045 [1] and, hence, diabetes care constitutes an important part of health care provision. Although diabetes can lead to a wide range of medical complications involving a range of different care professionals there is usually only one lead clinician that coordinates and provides the majority of this care. In the following text, this person is referred to as the primary diabetes care provider. In most cases, this primary diabetes care provider is a general practitioner or, in some countries, a specialist diabetes nurse. The primary diabetes care provider diagnoses the patient’s medical condition; discusses the treatment with the patient; provides drugs and any necessary medical equipment required for the treatment; counsels the patient; and supervises the patient’s adherence to the treatment regime. However, this can only be done if patients visit their primary care provider sufficiently frequently and diabetes care needs to be designed and organised to facilitate this. This, in turn, requires knowledge about the determinants of patient visits to the primary diabetes care provider.

Certainly, the most important determinant of visits to the primary diabetes care provider is cost: whether the patients must pay for the visit out of their own pocket; whether any costs are reimbursed through a health insurance scheme; or whether the system is financed through general taxation. There might, however, be other factors that affect the frequency of visits to the provider, which become more prominent once any cost issues are removed. These include the accessibility of the provider to the patient and the service quality of the provider. Important aspects of accessibility are travel distance and travel time from patient residence to provider location. Important aspects of the provider’s service quality, i.e. the extent to which the provider tries to respect the patients’ needs in all regards related to their interaction, are waiting time in the providers’ practice to see the clinician (i.e. the in-practice waiting time) and the quality of the provider’s communication with the patient. Long travel distances, long travel times, long in-practice waiting times (or combinations of these of these circumstances) might negatively affect the number of visits, whereas quality of communication might have a positive effect.

Several studies provide relevant evidence for the hypotheses formulated above. There are statistically significant negative associations between travel distance and visits to primary care providers [2], attendance at hospital emergency departments [3], frequency of visits to a dentist following traumatic dental injury [4], the decision to attend screening for gestational diabetes mellitus [5], the use of insulin [12] and glycaemic control [13 and 14] However, there was no statistically significant relationship with compliance for mammography [6] and health-related quality of life [7]. Other studies have produced statistically significant negative associations of travel time with the tendency to visit an endocrinologist [8], the likelihood of visiting a physician [9], the likelihood of attending a hospital [9], the likelihood of in-facility delivery [10], the frequency of pre-natal care visits [10, 11], and health-related quality of life [7]. However, there was no statistically significant association with the likelihood of visiting a general practitioner [15, 16], the likelihood of visiting a gynaecologist [16], compliance to mammography [6] and the delay in seeking care in case of a malaria infection [17]. Further studies have produced statistically significant associations of in-practice waiting time with frequency of physician visits [18] and health-related quality of life [7]. Moreover, some studies [19–21] show that quality of communication with the provider is positively associated with self-care.

At present, there is no study regarding the relationship of travel distance, travel time, in-practice waiting time and quality of provider-patient communication with the frequency of visiting the primary diabetes care provider. This study redresses that gap in the research literature with regard to care for diabetes type 2.

## Methods

### Study regions, study participants and study conduction

The investigation is based on the same data set that was used by Konerding et al. [7] to investigate the impact of travel distance, travel time and in-practice waiting time on health-related quality of life. These data were originally collected in the European FP-7 project ‘MANAGED OUTCOMES’ [22, 23], which was concerned with analysing health care provider networks in England, Finland, Germany, Greece, the Netherlands and Spain. In all of these countries, the majority of medical services are either reimbursed by health insurances or financed through taxation. However, there are differences between each country as to how the access to health care is regulated. In terms of the classification system of Reibling [24], England, the Netherlands and Spain are strong gatekeeping and low supply states, i.e. these states strongly regulate access to health care providers by having clinical expert ‘gatekeepers’ (general practitioners) decide which service should be provided to the patient. In turn, this means that these states – in theory at least - can have a lower supply of health care. Germany and Greece, by contrast, are weakly regulated and high supply states in the terminology of Reibling, i.e. these states have minimal gatekeeper control, which means that they have to provide a high supply of health care. Finally, in Reibling’s terminology, Finland is a ‘mixed regulation state’, i.e. this state regulates access to health service partially by gatekeeping and partially by cost sharing and has a moderate supply of health care [23].

In the MANAGED OUTCOMES project, health provider networks for different health conditions were investigated, including networks for providing type 2 diabetes care. For each country, one local network was selected and data were collected from the diabetes service locations in these networks. In England these locations were seven general practitioner practices associated with the Tower Hamlets Primary Care Trust in the east London Borough of Tower Hamlets. In Finland, these were the health centres of eight municipalities within Keski-Suomi (Central Finland). In Germany, these were one general practitioner and one diabetologist in the city of Bamberg, and two general practitioners and one diabetologist in the surrounding rural district of Bamberg. In Greece, these were five different institutions providing outpatient care for diabetes in the regional unit of Heraklion on the island of Crete. In the Netherlands, these were five general practitioner health centres in the region Nieuwe Waterweg Noord en Delft Westland Oostland. In Spain, this was one primary health area in the region of Valencia [7]. The main criterion for selecting the locations and health care providers was their willingness to cooperate in the study.

Part of the investigation comprised surveys of type 2 diabetes patients, which were undertaken with the assistance of the investigated primary diabetes care providers. These providers selected the patients to be approached for participation according to criteria defined by the researchers. Inclusion criteria for participants were (1) that they were being treated for type 2 diabetes by the health providers investigated in the project and (2) that they were at least 18 years old. Depending on the most feasible method for each provider, patients were contacted either by post or directly when they visited their primary diabetes care provider. Patients who participated in the survey completed their questionnaires on their own without any intervention by personnel from the service provider or the research team. Depending on the most feasible method for the particular provider, participants then returned their completed questionnaires either by post directly to the local project study centres, or to the care provider who then passed them on to the study centres. With the exception of Greece, all surveys were approved by national ethics committees. In Greece, the approval was granted by the relevant committee of the hospital. Data were collected between October 2011 and March 2012 [7].

### Questionnaire variables

The patient questionnaire applied in the survey contained questions addressing different topics. Two items addressed the mastery of the questionnaire language. Demographic data items included: sex, age and educational level of the patient (those leaving school directly after the minimum school leaving age of the country were classified as ‘low level’; those who had stayed in education beyond that were classified as ‘high level’). Health data items included the stage of diabetes, which was characterised by the three levels of treatment: (1) diet only (2) oral anti-diabetic agents but no insulin, and (3) insulin. The patients were also asked about any of five secondary complications they might have had arising from the diabetes: heart problems; previous stroke; problems with lower extremities; problems with sight; and kidney problems (either having had a kidney transplant or being dependent on dialysis). Other data referred to the diabetes service they attended: travel time; travel distance; in-practice waiting time; quality of communication; and the number of visits to the primary diabetes care provider within the last year (see Table 1).

**Table 1:**
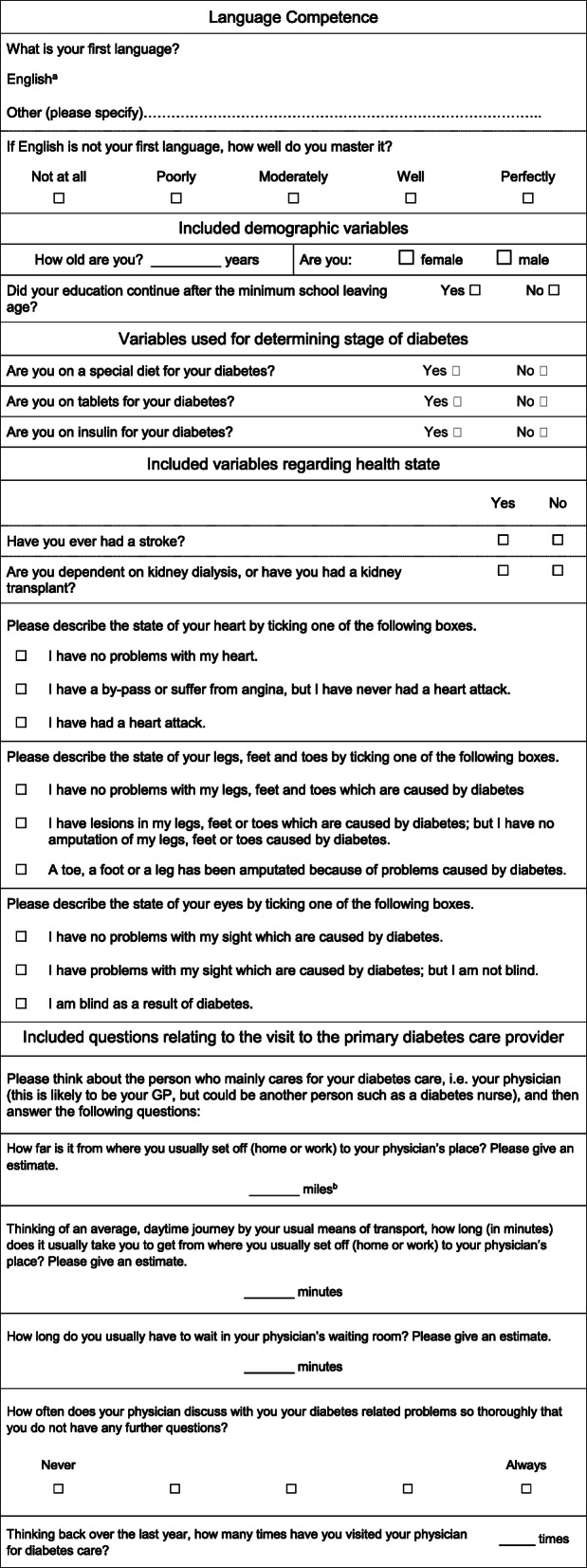
Questions in the questionnaire applied for the investigation

^a^For all other countries than England the respective national language was inserted

^b^For all other countries than England kilometres were asked. For the statistical analyses, the English miles were transformed into kilometres

The questions addressing mastery of the questionnaire language were included in order to determine the sample of participants included in the analyses, while all of the other variables were considered in the analyses themselves. In the following sections, these will be referred to as the ‘investigated variables’. According to their status in the statistical analyses, the investigated variables were subdivided into three categories. The number of provider visits, i.e. visit frequency, was classified as the criterion variable; travel time, travel distance, in-practice waiting time and quality of communication were classified as ‘focal predictor’ variables, and all remaining investigated variables were applied as control variables.

### Inclusion criteria

Patients were only included in the analysis if they had indicated that they mastered the questionnaire language well, and if they had given information about travel time; travel distance; in-practice waiting time, quality of communication; and the number of provider visits they had in the last year. Patients were excluded from analyses if data for more than two control variables were missing. The strict criteria with regard to missing data were chosen, because the number of missing data was assumed to reflect the thoroughness with which the respondent has answered the questionnaire. Accordingly, data of patients with more than two missing data were assumed to be less reliable and less valid than data of the other patients and it therefore seemed wise to exclude them from the analyses.

Patients were included independently of the number of visits to their primary diabetes care provider in the year preceding the survey, i.e. patients who had not visited at all during this time were also included. This was necessary because the relationship of the focal predictor variables with the number of visits was investigated and because ‘zero visits’ constituted a possible outcome. It could be argued that the answers given by patients with zero visits in the last year are less valid than the answers of regular visitors. However, even if they are less valid in the sense that they do not adequately describe the reality they can validly reflect what the patients think about the reality and it is this thinking which determines the behaviour [25].

### Statistical analyses

For the inclusion criteria, descriptive statistics were computed based on the total number of questionnaires distributed and, for the investigated variables, based on the questionnaires which met the inclusion criteria. Percentages were computed for dichotomous variables, and means, standard deviations, minima and maxima for continuous variables. This was undertaken for both the total sample and separately for the six region-specific samples. Regional differences regarding dichotomous variables were statistically tested using chi-square tests, and regional differences regarding continuous variables using Kruskal-Wallis tests.

The relationship of travel distance, travel time, in-practice waiting time and quality of communication with visits to the primary diabetes care provider was investigated using log-linear Poisson regression models [26] with frequency of visits as the criterion. Poisson regression models are specially designed for predicting count variables as for example the frequency of visits. The de-logarithmised coefficients estimated by these models reflect the proportion with which the predicted variable increases or, respectively, decreases when the corresponding predictor variable increases by one unit.

All focal predictor variables and all control variables were applied as predictor variables. All focal predictors were entered together into the same model as they were considered to reflect different aspects. This also applies to travel distance and travel time because travel time is not solely determined by travel distance, but also by factors such as the method of transport available to the patient and road congestion. Hence, both variables might have an independent effect on the visits to the diabetes care provider. Dummy-coded study regions were included to control for region-specific tendencies regarding provider visits. The status of diabetes and the five secondary complications of diabetes were included to control for the effect of health status because health status usually affects the number of health care provider visits [2–4, 8, 9, 15]. The three socio-demographic variables (age, gender and education) were included because these variables are known to be related to health status [27–30] and thus might help to control for those aspects of health status that are not reflected by diabetes status and secondary complications. Taken together, the demographic variables and the variables directly addressing health status can be regarded as variables covering the need for visits to the primary diabetes care provider.

The coefficients for all predictor variables were estimated and tested for deviation from zero. Fit of the models was investigated by comparing the complete model via a likelihood ratio test with only the criterion mean as predictor and by using the index proposed by Cameron and Windmeijer [31] (CW-index).^1^ The CW-index serves a similar purpose as the multiple R square in multivariate linear regression. This index is 0 when the model predicts the criterion as poorly as the criterion mean, and it is 1 when the model predicts the criterion optimally. The extent to which a specific predictor variable contributes to predicting visit frequency was assessed by subtracting the CW-index for a model without this variable from the CW-index for the complete model. To obtain a statistic that can be interpreted analogously to the percentage of variance exclusively explained by a specific predictor variable in a multivariate regression model, the resulting difference was multiplied by 100. In contrast to the coefficient belonging to a predictor variable, the statistic just described is independent of the unit with which the predictor variable is measured. Therefore, by means of this statistic, the contributions of different predictor variables can be compared.

The correlations between the focal predictors were computed and, to investigate the influence of these correlations on the results, four additional models were computed in each of which a different focal predictor was omitted.

To investigate the stability of the results across the six study regions, the interactions between the focal predictor variables and the study regions were analysed. The statistical significance for the interactions with a specific focal predictor was tested via a likelihood ratio test by comparing a model containing all predictor variables and all investigated interactions with a model from which the interaction terms for the respective focal predictor variable had been removed. In addition, the contribution of the interaction for explaining the visit frequency was investigated using CW-indices in an analogous manner as described above for the main effects. To investigate the extent to which possible interactions can be attributed to the countries’ health care systems, analogue computations were performed after grouping the study regions according to the Reibling categories assigned to the corresponding country [23, 24] (see above).

To check the generalisability of the results, all analyses were performed in two variants: 1) for all included participants (i.e. persons who had up to two missing data items for the control variables), and 2) for participants with complete data for all investigated variables. For the analysis referring to participants with missing values, the missing values were imputed by the study region-specific means. As data possessing less than interval scale level were dummy coded, the study region-specific relative frequencies of persons belonging to the respective category were taken for imputing missing values of these data.

## Results

Altogether 6245 questionnaires were distributed of which 1638 (26.2%) were returned and 1086 (17.4%) met the inclusion criteria (see Table 2). The proportions of included questionnaires vary from 6.4% for England to 47.2% for Germany (see Table 2). The proportion of included questionnaires is smallest in England because about 40% of all respondents in this sample were of Bangladeshi ethnicity who, due to lower levels of stated proficiency in the English language, did not meet the inclusion criteria. In total 829 (13.3%) of the included questionnaires had no missing data for the investigated variables (see Table 2). There are study region differences with regard to all investigated variables except for gender, and for kidney problems (see Table 3). In the sample of all included patients, the numbers of visits per year range from zero to 40 with a mean of 3.9 and a standard deviation of 3.1 (see Table 3).

**Table 2:** General information about the sample

	English region	Finnish region	German region	Greek region	Dutch region	Spanish region	Total sample
Statistics determining the study sample^a^							
Questionnaires distributed	3343	436	462	600	779	625	6245
Questionnaires returned	475 (14.2%)	183 (42.0%)	286 (61.9%)	179 (29.8%)	400 (51.3%)	115 (18.4%)	1638 (26.2%)
Sufficient language competence^b^	313 (9.4%)	183 (42.0%)	282 (61.0%)	179 (29.8%)	387 (49.7%)	115 (18.4%)	1459 (23.4%)
Sufficient data^c^	286 (8.6%)	135 (31.0%)	221 (47.8%)	153 (25.5%)	304 (39.0%)	71 (11.4%)	1170 (18.7%)
Participants included	213 (6.4%)	135 (31.0%)	218 (47.2%)	153 (25.5%)	296 (38.0%)	71 (11.4%)	1086 (17.4%)
Complete data^d^	160 (4.8%)	103 (23.6%)	172 (37.2%)	110 (18.3%)	227 (29.1%)	57 (9.1%)	829 (13.3%)

^a^ Percentages refer to questionnaires distributed

^b^ The English sample contained a large proportion of persons with Bangladeshi ethnicity who had lower levels of stated proficiency in the English language

^c^ Participants with complete data for the criterion variable and the four focal predictor variables and with at most two missing values for the control variables

^d^ Participants with complete data for all investigated variables

**Table 3:** Distributions of the investigated variables

	English region	Finnish region	German region	Greek region	Dutch region	Spanish region	Total sample	Region differences^a^
Control variables^b^								
Gender								
Valid data	209	132	217	153	290	70	1071	
Male	64.6%	65.2%	53.9%	58.2%	59.3%	55.7%	59.6%	n.s.^c^
Age in years								
Valid data	211	133	218	153	294	71	1080	
Mean (SD)	63.0 (11.9)	63.5 (9.6)	65.7 (11.2)	66.0 (10.6)	65.0 (10.0)	69.3 (11.4)	65.0 (10.9)	*p* < 0.001^d^
Education								
Valid data	191	129	211	150	287	68	1036	
High	39.8%	59.7%	66.4%	27.3%	76.3%	26.5%	55.1%	*p* < 0.001^c^
Stage of diabetes								
Valid data	204	124	207	125	276	63	999	
Diet only	13.7%	3.2%	7.7%	1.6%	19.9%	4.8%	10.8%	*p* < 0.001^c^
Oral, no insulin	65.7%	69.4%	58.9%	68.8%	65.9%	63.5%	65.1%	
Insulin	20.6%	27.4%	33.3%	29.6%	14.1%	31.7%	24.1%	
Heart problems								
Valid data	207	126	193	144	284	70	1024	
Yes	25.1%	19.0%	21.8%	30.6%	14.8%	18.6%	21.2%	*p* < 0.01^c^
Previous stroke								
Valid data	212	135	217	151	289	70	1074	
Yes	7.5%	2.2%	10.6%	6.0%	4.5%	5.7%	6.3%	*p* < 0.05^c^
Problems with lower extremities								
Valid data	206	134	215	148	283	71	1057	
Yes	17.0%	12.7%	26.0%	12.2%	5.3%	15.5%	14.4%	*p* < 0.001^c^
Problems with sight								
Valid data	210	133	216	151	292	70	1072	
Yes	24.3%	10.5%	17.6%	15.9%	8.6%	28.6%	16.0%	*p* < 0.001^c^
Problems with kidney								
Valid data	207	133	212	145	286	71	1054	
Yes	0.5%	0.0%	0.9%	0.7%	0.0%	0.0%	0.4%	n.s.^c^
Focal predictors^e^								
Travel distance to provider in kilometres								
Mean (SD)	1.6 (2.9)	9.2 (10.7)	5.7 (6.1)	15.8 (20.6)	1.9 (1.8)	1.5 (1.3)	5.4 (10.4)	*p* < 0.001^d^
Travel time to provider in minutes								
Mean (SD)	14.6 (9.9)	16.7 (11.5)	13.1 (9.2)	28.8 (25.9)	7.7 (5.1)	14.4 (9.8)	14.7 (14.2)	*p* < 0.001^d^
In-practice waiting time in minutes								
Mean (SD)	18.5 (13.9)	13.7 (10.3)	43.6 (32.5)	52.8 (41.1)	12.9 (19.6)	38.2 (30.0)	27.5 (30.1)	*p* < 0.001^d^
Quality of communication^f^								
Mean (SD)	3.9 (1.2)	4.2 (1.0)	4.2 (1.1)	3.9 (1.3)	4.4 (1.0)	3.5 (1.5)	4.1 (1.2)	*p* < 0.001^d^
Criterion variable^e^								
Numbers of physician visits per year								
Mean (SD)	3.3 (3.0)	2.5 (1.4)	4.6 (4.0)	4.9 (4.3)	3.7 (1.3)	4.6 (5.9)	3.9 (3.3)	*p* < 0.001^d^

^a^ *n.s.* not significant

^b^ Valid data vary due to missing values

^c^ Pearson’s chi-square test

Kruskal-Wallis test^e^

^e^ Valid data are identical with the numbers of participants included (see Table 1)

^f^ Coded from 1 for worst quality to 5 for best quality

The results of the Poisson regressions for predicting yearly provider visits are largely the same when patients with up to two missing values and when only patients with complete data are included (See Table 4). In both cases, the model predicts visit frequency essentially better than the mean (in both cases: *p* < 0.001). When patients with missing data are considered, the CW-Index is 0.19. When only patients with complete data are considered, it is 0.18. The control variables contribute largely to the model prediction (See Table 4). There are statistically significant negative relationships of travel distance and in-practice waiting time with visit frequency. For travel distance, this relationship is large; for in-practice waiting time it is small. The relationship between travel time and visit frequency is not statistically significant. Quality of communication has a very strong statistically significant positive relationship with visit frequency (See Table 4).

**Table 4:** Prediction of yearly patient visits to the primary diabetes care provider

	Participants with up to two missing values (*n* = 1086)			Patients with complete data (*n* = 829)		
	Parameter^a^	%-change^b^	%-cont^c^	Parameter^a^	%-change^b^	%-cont^c^
Constant	0.540***			0.577***		
Control variables						
Finnish region^d^	− 0.218**	−19.59	7.77	− 0.248**	−21.94	8.64
German region^d^	0.361***	43.48		0.330***	39.08	
Greek region^d^	0.541***	71.77		0.545***	72.46	
Dutch region^d^	0.163**	17.70		0.163**	17.74	
Spanish^d^	0.411***	50.83		0.515***	67.30	
Male gender	− 0.001	− 0.10	0.03	− 0.001	− 0.12	0.03
Age in years	0.043	4.39	0.08	0.036	3.69	0.06
High education	− 0.059	−5.73	0.12	−0.051	−4.99	0.10
Oral medicaments, no insulin^e^	0.107	11.29	0.80	0.122	12.96	0.53
Insulin^e^	0.241***	27.25		0.208**	23.16	
Heart problems	0.044	4.50	0.06	0.058	5.95	0.10
Previous stroke	0.166**	18.06	0.34	0.109	11.47	0.12
Extremities problems	0.332***	39.38	2.37	0.330***	39.05	2.36
Sight problems	−0.017	−1.69	0.01	− 0.023	−2.26	0.01
Kidney problems	−0.938**	−60.86	0.53	−0.856**	−57.53	0.58
Focal predictors						
Travel distance (km)	−0.011***	−1.09	1.06	−0.010***	−0.99	0.97
Travel time (minutes)	0.002	0.20	0.09	0.003	0.30	0.20
In-practice waiting time (minutes)	−0.001*	− 0.10	0.21	− 0.002*	− 0.16	0.30
Quality of communication^f^	0.127***	13.54	3.71	0.121***	12.85	3.26

^a^ Asterisks behind the values symbolize statistical significances for deviation from zero with * = *p* < 0.05, ** = *p* < 0.01, and *** = *p* < 0.001

^b^ Percentage of increase or, respectively, decrease of yearly visits when the respective predictor variable increases for one unit

^c^ Contribution of the respective predictor variable to the prediction of the complete model (one hundred times the difference between the CW-Indices for the complete model and the models without the respective predictor variable)

^d^ The reference category is England

^e^ The reference category is treatment only by diet

^f^ Coded from 1 for worst quality to 5 for best quality

The correlations between the four focal predictors are largely the same for patients with up to two missing values and for patients with complete data. Quality of communication does not correlate with travel distance and travel time. In the remaining cases, the correlations deviate from zero on the 0.001 level. For travel distance and travel time, the correlations are 0.696 for patients with up to two missing values and 0.698 for patients with complete data. For travel distance and in-practice waiting time, these values are 0.215 and 0.254. For travel time and in-practice waiting time, they are 0.273 and 0.286. For in-practice waiting time and quality of communication, they are − 0.132 and − 0.150. When travel time or in-practice waiting time are removed from the model, the pattern for the coefficients of the remaining three focal predictors stays unaltered both for patients with up to two missing values and for patients with complete data. When travel distance is removed, the coefficient pattern for the remaining three variables stays largely the same for patients with complete data, but for patients with up to two missing values a slightly negative statistically significant effect of travel time comes into existence. When quality of communication is removed, the results are largely the same for patients with up to two missing data and for patients with complete data. In both cases, the results for travel distance and in-practice waiting time stay largely unaltered, but a slightly positive statistically significant effect of travel time arises, i.e. the visit frequency increases with increasing travel time.

When terms for the interaction between study regions and focal predictors are added, the model predicts visit frequency essentially better than the mean (for patients with up to two missing values and for patients with complete data: *p* < 0.001). The CW-indices increase essentially, from 0.18 to 0.24 for patients with up to two missing values and from 0.19 to 0.25 for patients with complete data. The patterns of coefficients and the corresponding statistical significances are largely the same for both groups of patients. The interaction of study regions with travel time is not statistically significant; the interactions with the remaining three focal predictors, however, are highly significant (see Table 5). For travel distance and in-practice waiting time there are even statistically significant effects in opposite directions. To be specific, in both patient groups, visit frequency increases significantly with travel distance in the English region, whereas the opposite is true in the Greek region. In a similar way, visit frequency increases significantly with in-practice waiting time in the Spanish region, whereas the opposite is true for the Finnish and Greek regions. In contrast, visit frequency increases with quality of communication in all regions and, in most cases, this increase is statistically significant. The study regions only differ with regard to the extent of the increase. It is smallest for the Greek and largest for the Spanish region (see Table 5).

**Table 5:** Prediction of yearly patient visits to the primary diabetes care provider; country specific coefficients for focal predictors^a^

	English region	Finnish region	German region	Greek region	Dutch region	Spanish region	Country differences^b^
Participants with up to two missing values (*n* = 1086)							
Travel distance in kilometres	0.027**	0.005	−0.009	−0.015***	− 0.005	−0.110*	1.02***
Travel time in minutes	0.004	0.000	−0.002	0.005*	−0.003	0.012*	0.24
In-practice waiting time in minutes	−0.002	−0.014*	0.001	−0.006***	− 0.001	0.006***	2.16***
Quality of communication^c^	0.163***	0.133*	0.142***	0.030	0.068*	0.315***	1.56***
Patients with complete data (*n* = 829)							
Travel distance in kilometres	0.025*	0.006	−0.003	−0.014***	0.002	−0.112	1.15**
Travel time in minutes	0.004	0.001	0.001	0.006**	−0.004	0.015*	0.32
In-practice waiting time in minutes	−0.009*	−0.020*	0.002	−0.009***	− 0.007	0.006***	4.04***
Quality of communication^c^	0.127**	0.190*	0.159***	0.004	0.068	0.228***	1.12**

^a^ Parameters of Poisson regression when the respective country is taken as reference for the interactions. Statistical significances for deviation from zero are symbolised with * = *p* < 0.05, ** = *p* < 0.01, and *** = *p* < 0.001

^b^ Contribution of the respective interactions to the prediction of the model with interactions for all four focal predictors, i.e. one hundred times the difference between the CW-Indices for the model with interactions for all focal predictors and the models without interactions for the respective focal predictor. The statistical test was performed using a likelihood ratio test between both models. Statistical significances are symbolised with * = *p* < 0.05, ** = *p* < 0.01, and *** = *p* < 0.001

^c^ Coded from 1 for worst quality to 5 for best quality

When the interaction terms for study regions are replaced by interaction terms with the Reibling categories, the model also predicts visit frequency better than the mean (for patients with up to two missing values and for patients with complete data: *p* < 0.001). However, the CW-indices are only slightly higher than the corresponding indices of the models without any interaction terms. These indices are 0.20 for both patient groups. Apart from this, the general pattern of results is similar to the pattern of results for interactions with study regions. There are statistically significant interaction effects with travel distance and in-practice waiting time in both patient groups and a statistically significant interaction effect for quality of communication for patients with up to two missing values (see Table 6). There are coefficients in opposite direction for travel distance and in-practice waiting time. However, the only coefficients that are statistically significant are negative (see Table 6). This applies to travel distance and to in-practice waiting time in both patient groups (see Table 6), i.e. in these cases visit frequency decreases with increasing travel distance and in-practice waiting time. For quality of communication, all coefficients are positive and statistically significant, i.e. visit frequency increases with quality of communication (see Table 6).

**Table 6:** Prediction of yearly patient visits to the primary diabetes care provider; Reibling category specific coefficients for focal predictors^a^

	Strong gatekeeping, low supply (England, the Netherlands, Spain)	Mixed regulation (Finland)	Weakly regulated, high supply (Germany, Greece)	Differences between categories^b^
Participants with up to two missing values (*n* = 1086)				
Sample sizes	580	135	371	
Travel distance in kilometres	0.011	0.005	−0.014***	0.54**
Travel time in minutes	0.002	0.000	0.003	0.01
In-practice waiting time in minutes	0.001	−0.015*	−0.002**	0.55**
Quality of communication^c^	0.161***	0.132*	0.085***	0.29*
Patients with complete data (*n* = 829)				
Sample sizes	444	103	282	
Travel distance in kilometres	0.008	0.006	−0.014***	0.59**
Travel time in minutes	0.002	0.000	0.004*	0.03
In-practice waiting time in minutes	0.001	−0.020*	−0.002**	0.67**
Quality of communication^c^	0.137***	0.187*	0.092***	0.15

^a^ Parameters of Poisson regression when the respective category is taken as reference for the interactions. Statistical significances for deviation from zero are symbolised with * = *p* < 0.05, ** = *p* < 0.01, and *** = *p* < 0.001

^b^ Contribution of the respective interactions to the prediction of the model with interactions for all four focal predictors, i.e. one hundred times the difference between the CW-Indices for the model with interactions for all focal predictors and the models without interactions for the respective focal predictor. The statistical test was performed using a likelihood ratio test between both models. Statistical significances are symbolised with * = *p* < 0.05, ** = *p* < 0.01, and *** = *p* < 0.001

^c^ Coded from 1 for worst quality to 5 for best quality

## Discussion

### Discussion of the study methodology

#### Generalisability

The study regions and the institutions within the study regions were selected because of their willingness to cooperate in the research project. Hence, the study regions cannot be taken as prototypical examples of all regions in the corresponding country. For each institution, all eligible patients were approached to participate in the survey, but only a subset of them responded and some of those responders had to be excluded for reasons of insufficient mastery of the questionnaire language or insufficient provision of valid data. Hence, the samples finally included are not even representative of the populations of eligible patients within the study regions. The persons represented by these samples are cooperative and thorough, and native or well-integrated citizens.

Regarding interpretation of the results, the selection bias in the participants implies that the results should only be referred to the native and the well-integrated citizens in the study region. This is so, because the study gives no information as to what would hold for non-native or not well-integrated citizens. As far as the selection bias is caused by willingness to cooperate and thoroughness, the situation is slightly different. Participants who have answered all questions can be considered to be more cooperative and more thorough than those who left out one or two questions. Hence, the sensitivity analyses indicate how willingness to cooperate and thoroughness might affect the results. When the results for patients with up to two missing values are largely the same as those for patients with complete data then this indicates that the effect found in the analysis might hold in the region specific population of all native or well-integrated patients with type 2 diabetes.

The manner in which the study regions have been selected implies that the results found in the region cannot readily be generalised to the corresponding country. However, country-specific characteristics constitute one possible explanation of similarities and differences between the results from different study regions. An alternative explanation is that these similarities and differences are caused by other study region characteristics that are not representative of the corresponding countries. Comparison of the results from across the different study regions gives some information regarding the generalisability of results. Where results differ between the regions, this shows that these results depend on special features of the study regions and/or the corresponding patients. However, when the results are stable across the regions this indicates that similar results might be found in different regions. In this regard, performing a study with data from six different European provider networks with different participant samples constitutes an important strength of the study.

#### Study design

The study presented here is a cross-sectional study and so it is unclear as to whether the relationships found are actually causal and, if so, which variable is the cause and which the effect. To test this, a randomised controlled trial (RCT) would be necessary and such a study design is usually not feasible in this context. Therefore, health care design is dependent on empirical studies using weaker designs than RCTs (such as cross-sectional studies). To assess whether the relationships found in such studies are causal and what the direction of causality might be, further knowledge about the target of investigation must also be considered. The best that cross-sectional designs can produce are empirically well-substantiated hypotheses regarding causal relationships and the results presented here are to be understood precisely in this way. It is up to the health care designers to decide whether they consider the empirical substantiation as sufficient to base interventions on them. When these interventions are evaluated using RCTs, these evaluation studies constitute tests of the causality assumptions on which the interventions are based.

#### Multiple testing

As the effects of several different variables were tested at the same time, concerns regarding an increased probability of false positive results due to multiple testing might arise. However, as the statistical tests were performed as parts of multivariate regression models and as these models were highly statistically significant the different positive results of the significance tests cannot simply be explained by chance.

### Interpretation of the study results

For the data combined across the six study regions a clear picture seems to emerge. The pattern of results is largely the same for patients with up to two missing data and for patients with complete data. Hence, the results could be generalised to all native or well-integrated patients in the study regions. The results indicate a moderate negative effect of travel distance on visits to the diabetes care provider, no effect due to travel time, a very slight effect of in-practice waiting time and a very strong effect due to quality of communication (see Table 4). The analyses where focal predictors were alternatively removed from the model corroborate the interpretation that it is travel distance and not travel time that negatively affects visit frequency for the aggregated data of the six study regions. This corresponds to previous research findings as there were more non-significant results in studies addressing the effect of travel time [6, 15–17] than in studies addressing the effect of travel distance [6]. However, once again, quality of communication has the by far largest effect. An increase of 1 on the five-categorical scale is associated with a 13.54% increase of visit frequency. Consequently, an increase from the lowest to the highest category would lead to a 54.16% increase of visit frequency.

When region-specific coefficients for the four focal predictors are computed, the clear and simple picture disappears. Except for travel time, there are large differences between the study regions. For travel distance and for in-practice waiting time there are even statistically significant effects in opposite directions. This implies that there are statistically significant effects that are inconsistent with previous results and with intuition for each of the two variables. For travel distance, this holds for the English region, where this variable has a positive effect on visit frequency and, for in-practice waiting time, this holds for the Spanish region, where this variable has a positive effect (see Table 5). The most probable reason for the effect in the English region is that, in this region, travel distance is correlated with a further variable that has a positive impact on visit frequency and that is not contained in the regression model. For the Spanish region there seems to be something analogue for in-practice waiting time. The point is that the results suggest that travel distance and in-practice waiting time affect visit frequencies differently in different contexts. Although there is also an interaction effect for quality of communication, there is an essential commonality between all six study regions: visit frequencies always increase with quality of communication. In nine of 12 cases, this relationship is even statistically significant (see Table 5). Hence, there is a high probability that effects of this kind will also be found in regions that have not been investigated in this study.

Future development of health care provision might benefit from understanding reasons why most of the focal predictors have different relationships to visit frequencies in the different regions. One reason might be that the impact of the focal predictors is moderated by the country-specific regulations regarding access to health care providers. This hypothesis was tested by grouping the regions according to their country-specific regulations as described by the classification system of Reibling and by repeating the computations with this grouping. With this computation, the interaction effects strongly decrease, if they remain at all. This suggests that country-specific regulations regarding access to health care are not an important reason for different effects of the focal predictors in the different study regions. There must be different characteristics of the study region and/or the study samples that are responsible for the interaction effects and identifying these would be a worthwhile subject for further research.

As the increase of visit frequency with quality of communication is the only stable effect, the question rises as to whether this relationship is causal with quality of communication as cause and visit frequency as the effect. As people generally tend to seek out other people with whom they have good communication, so it might be reasonable to interpret the relationship found here in this way. At least, this might be enough to justify the recommendation to increase quality of communication if there is an aim to increase the frequency of visits to the diabetes care provider.

## Conclusions

Travel time, when controlled for travel distance, appears to have no impact on the frequency of visits to the diabetes care provider. For travel distance and in-practice waiting time, the effects are slight and inconsistent across study regions. In contrast, quality of communication has a strong positive effect on visit frequency. The strength of this relationship varies across study regions, but such a relationship was apparent in all of them, at least at a descriptive level. Therefore, if one seeks to increase the frequency of visits to the diabetes care provider increasing the quality of the provider’s communication with the patient would be highly recommended.

## Data Availability

The datasets used and/or analysed during the current study are available from the corresponding author on reasonable request.

## Abbreviations

CW-Index: Index proposed by Cameron and Windmeijer for testing the fit of log-linear Poisson regression models
RCT: Randomised controlled trial

## Footnotes

1 Following a convention reported by Greene [26] ln (0) was set equal to zero when CW-indices were computed.
